# The influence of HIV‐related stigma on PrEP disclosure and adherence among adolescent girls and young women in HPTN 082: a qualitative study

**DOI:** 10.1002/jia2.25463

**Published:** 2020-03-06

**Authors:** Jennifer Velloza, Nomhle Khoza, Fiona Scorgie, Miria Chitukuta, Prisca Mutero, Kudzai Mutiti, Nomvuyo Mangxilana, Lumka Nobula, Michelle A Bulterys, Millicent Atujuna, Sybil Hosek, Renee Heffron, Linda‐Gail Bekker, Nyaradzo Mgodi, Mike Chirenje, Connie Celum, Sinead Delany‐Moretlwe, for the HPTN 082 study group

**Affiliations:** ^1^ University of Washington Seattle WA USA; ^2^ Wits Reproductive Health & HIV Institute (Wits RHI) Johannesburg South Africa; ^3^ Clinical Trials Research Centre University of Zimbabwe College of Health Sciences Harare Zimbabwe; ^4^ Desmond Tutu HIV Foundation Cape Town South Africa; ^5^ Stroger H. Hospital of Cook County Chicago IL USA; ^6^ Faculty of Health Sciences Institute of Infectious Disease and Molecular Medicine University of Cape Town Cape Town South Africa; ^7^ Faculty of Health Sciences University of the Witwatersrand Johannesburg South Africa

**Keywords:** Stigma, disclosure, HIV, pre‐exposure prophylaxis, Africa, women

## Abstract

**Introduction:**

Stigma and disclosure concerns have been key barriers to oral pre‐exposure prophylaxis (PrEP) adherence for African adolescent girls and young women (AGYW) in efficacy trials. We aimed to understand the impact of these factors among African AGYW in an open‐label PrEP study.

**Methods:**

HPTN 082 was an open‐label PrEP study among AGYW (ages 16 to 24) in Harare, Zimbabwe, and Cape Town and Johannesburg, South Africa from 2016 to 2018. Women starting PrEP were randomized to standard adherence support (counselling, two‐way SMS, monthly adherence clubs) or standard support plus drug‐level feedback. Serial in‐depth interviews were conducted among 67 AGYW after 13‐week and 26‐week study visits to explore experiences of stigma, disclosure and PrEP adherence. We analysed data by coding transcripts and memo‐writing and diagramming to summarize themes.

**Results:**

AGYW described stigma related to sexual activity (e.g. “people say I'm a prostitute”) and being perceived to be living with HIV because of taking antiretrovirals (e.g. “my husband's friends say I'm HIV infected”). Participants who anticipated stigma were reluctant to disclose PrEP use and reported adherence challenges. Disclosure also resulted in stigmatizing experiences. Across all sites, negative descriptions of stigma and disclosure challenges were more common in the first interview. In the second interview, participants often described disclosure as an “empowering” way to combat community‐level PrEP stigma; many said that they proactively discussed PrEP in their communities (e.g. became a “community PrEP ambassador”), which improved their ability to take PrEP and encourage others to use PrEP. These empowering disclosure experiences were facilitated by ongoing HPTN 082 study activities (e.g. counselling sessions, adherence clubs) in which they could discuss PrEP‐related stigma, disclosure and PrEP adherence issues.

**Conclusions:**

Stigma and disclosure challenges were initial concerns for African AGYW newly initiating PrEP but many were empowered to disclose PrEP use over their first six months of PrEP use, which helped them cope with stigma and feel more able to take PrEP regularly. PrEP programmes can foster disclosure through community and clinic‐based discussion, adherence clubs and activities normalizing sexual behaviour and PrEP use, which can reduce stigma and improve PrEP adherence and thus effectiveness.

## 1 Introduction

Daily oral pre‐exposure prophylaxis (PrEP) with emtricitabine/tenofovir disoproxil fumarate (FTC/TDF) is >90% effective in preventing HIV when taken consistently 1, 2, 3, 4, 5, 6. Adherence can be challenging, however, and suboptimal adherence in efficacy trials was largely attributed to concerns around product safety, effectiveness, and anticipated and experienced PrEP stigma 5, 7, 8. “Anticipated stigma” includes fears about others’ negative reactions to PrEP, while “experienced stigma” describes first‐hand experience of stigma resulting from PrEP use 7, 8, 9, 10. PrEP has been stigmatized because antiretrovirals with the same appearance are used for HIV treatment, and PrEP users may be mistakenly labeled as “HIV positive” 11, 12, 13. PrEP may also be seen as promoting sexual promiscuity, leading to stigma related to norms around sexuality in adolescent girls and young women (AGYW) 11, 14, 15.

PrEP demonstration programmes have found that women can initially adhere to PrEP 16, 17, 18, but adherence wanes over time with stigma remaining a barrier to sustained PrEP use during periods of HIV risk 16, 17, 18. Stigma can be particularly high in contexts where community awareness about PrEP is low, cultural norms dictate that unmarried women should not be sexually active or should practice monogamy, and PrEP was initially prioritized for “high‐risk” key populations, including female sex workers (FSWs) 14, 19, 20, 21. Quantitative analyses among men who have sex with men (MSM) in the United States and Africa and heterosexual women in the United States have found that stigma significantly reduces PrEP interest, uptake and adherence 14, 22, 23, 24, 25, 26, 27, 28. Stigma may also influence African women's PrEP adherence as shown by qualitative work conducted among AGYW and FSWs that describes women's need to discreetly use HIV prevention products and miss doses because of concerns around stigma 18, 19, 27, 28, 29, 30.

The influence of stigma on disclosure and PrEP use has not been well‐explored among AGYW in sub‐Saharan Africa. Moreover experiences with PrEP‐related stigma and disclosure could dynamically shift over time as community knowledge of PrEP increases 20, 31, 32. We describe experiences of and changes in PrEP‐related stigma, disclosure and adherence over time using qualitative serial, in‐depth interview data from AGYW enrolled in the open label PrEP study, HPTN 082, in South Africa and Zimbabwe.

## 2 Methods

### 2.1 Study design and participants

The HIV Prevention Trials Network (HPTN) 082/HERS study was a randomized, open‐label trial with AGYW who were offered daily oral FTC/TDF PrEP. The study enrolled sexually active AGYW, ages 16 to 25, in Johannesburg and Cape Town, South Africa and Harare, Zimbabwe between 2016 and 2018. Eligible women were HIV‐negative, literate in English, isiXhosa, isiZulu, SeSotho or Shona, and at high risk of HIV as determined by an empiric risk score 33.

Participants were offered PrEP at enrollment. Those who accepted PrEP were randomized in a 1:1 ratio to one of two adherence interventions: (1) standard adherence support including adherence counselling sessions and educational brochures with information about PrEP, weekly short message service (SMS) reminders, and monthly in‐person adherence support clubs, or (2) standard adherence support plus counselling based on PrEP drug levels measured at weeks 4 and 8 (“drug‐level feedback”). Counselling sessions lasted approximately 30 minutes and focused on PrEP adherence problem‐solving including role plays around disclosing PrEP use to others as participants felt ready. Monthly adherence clubs lasted approximately one hour and included discussion of facilitators and barriers to PrEP use. Follow‐up visits occurred at four, eight, thirteen, twenty‐six, thirty‐nine and fifty‐two weeks post‐enrollment and included HIV testing and PrEP refills. Participants who initially declined PrEP at enrollment were followed on the same schedule and were offered PrEP during follow‐up visits.

### 2.2 Qualitative recruitment

Up to 25 participants were purposively recruited for qualitative interviews per study site. We hypothesized that this sample size would be adequate to achieve saturation of key themes based on qualitative work investigating similar issues of stigma and disclosure among new PrEP users 18, 34, 35. Participant selection was stratified into three groups at each site: those who accepted PrEP and adhered well by week 4, those who accepted PrEP but had difficulty adhering by week 4, and those who declined PrEP through the first 12 weeks of follow‐up. PrEP acceptance was assessed via PrEP dispensation records and PrEP adherence was defined using plasma drug level data (with tenofovir levels >40 ng/mL considered “high adherence”) 36, 37. Adherence at weeks 13 and 26 was also assessed with dried blood spot data, with tenofovir diphosphate levels (TFV‐DP) ≥700 fmol/punch consistent with four or more doses per week 38. At each site, two to four participants were selected as “interesting cases” (e.g. reported social harms, had a protocol‐defined PrEP discontinuation due to pregnancy, creatinine levels, potential HIV seroconversion) based on experiences prior to week 13. Participants were recruited by phone or during HPTN 082 visits and were asked to complete interviews after 13‐week and 26‐week study visits.

### 2.3 Data collection

Semi‐structured, in‐depth interview guides were developed based on the literature and experiences with PrEP delivery. The 13‐week guide included questions related to community knowledge about PrEP, motivations to take PrEP, facilitators and barriers to PrEP uptake and adherence, and PrEP disclosure. The 26‐week interview covered similar topics and asked about changes experienced since the prior interview.

Interviews were carried out by female qualitative researchers, conducted in participants’ preferred language, audio‐recorded and approximately 45 minutes. Recordings were transcribed and translated into English. Transcripts were reviewed by the sites’ qualitative teams to check for accuracy. Participants received reimbursement for their time and travel to the sites for interviews (10 to 15 USD).

### 2.4 Data analysis

For our thematic analysis, we used the constant comparative method developing an initial codebook from themes in the data and refining the codebook during the coding process 39, 40. Transcripts were imported into NVivo (QSR International, Melbourne, Australia) and each transcript was coded by one member of the study team (JV, NK, FS, MB, PM, LM, NM, MA or SH). One member of the study team (FS) reviewed coding for 20% of the transcripts and coding disagreements were resolved through discussion. We reviewed the codes, wrote analytic memos and used diagramming techniques (e.g. drawing out conceptual models to display relationships between codes and changes to codes over time) to identify key themes across transcripts and demographic characteristics 41. The analytical work included in‐person workshops with study team members from the three sites and group discussions about emerging themes.

We also utilized a case‐specific analytic approach to identify changes in participants’ narratives over time 42, 43. After reviewing interview transcripts and coded text, one team member (JV) developed a participant‐level matrix of key themes from the 13‐week and 26‐week interviews. We examined themes in the matrix by site, demographics and PrEP use and wrote summary memos on longitudinal patterns.

### 2.5 Ethical statement

This study received ethical approval from review boards at the University of California – San Francisco, University of Washington, and each study site. All participants provided written informed consent in their preferred language. The protocol was registered at http://Clinicaltrials.gov (NCT02732730).

## 3 Results

### 3.1 Participant characteristics

Sixty‐seven AGYW (of 451 HPTN 082 participants) were interviewed (Table 1). At enrollment, participants had median age of 21 and 97.0% completed some secondary school or higher, similar to the demographics of the HPTN 082 sample 44. Fifty‐seven participants completed both interviews, two participants completed only their second interview and eight participants completed only their first interview. Among these eight participants, four completed all other study procedures but refused a second interview and four discontinued HPTN 082 participation early. All Harare participants were retained through both interviews, but only 66.7% of Cape Town and 82.3% of Johannesburg participants completed both interviews. There were no differences in demographics between participants who completed both interviews and those who did not, but a larger proportion of those who were not retained reported a primary partner at enrollment (100% vs. 67% among those retained) and a higher proportion declined PrEP at Week 13 (12.5% vs. 4.2% in those who were retained for both interviews).

**Table 1:** Characteristics of the HPTN 082 qualitative study sample (N = 67, unless otherwise indicated)

Characteristic	Site demographics^a^		
	Harare N = 25 (37.3)	Cape Town N = 20 (29.9)	Johannesburg N = 22 (32.8)
Age^b^	20 (19 to 22)	20 (19 to 20)	24 (22 to 25)
Education (N = 66)^b^			
Completed secondary school or higher	19 (76.0)	9 (47.4)	20 (90.9)
Some secondary school	5 (20.0)	11 (57.9)	1 (4.5)
Completed primary school	1 (4.0)	0 (0.0)	1 (4.5)
Employment status (N = 66)^b^			
Employed	8 (32.0)	1 (5.0)	2 (9.5)
Unemployed	12 (48.0)	6 (30.0)	6 (28.6)
Current student (either secondary or tertiary education)	5 (20.0)	13 (65.0)	13 (61.9)
Relationship status^b^			
Single, no sexual partners	8 (32.0)	0 (0.0)	1 (4.5)
Dating (at least one sexual partner but casual)	0 (0.0)	0 (0.0)	4 (18.2)
In a relationship (at least one main serious partner)	10 (40.0)	20 (100.0)	17 (77.3)
Married	7 (28.0)	0 (0.0)	0 (0.0)
Living with^b^			
Parents (with or without siblings or own children)	10 (40.0)	18 (90.0)	19 (86.4)
Partner (with or without other roommates or own children)	11 (44.0)	0 (0.0)	1 (4.5)
Other	3 (12.0)	1 (5.0)	1 (4.5)
Alone	1 (4.0)	1 (5.0)	1 (4.5)
PrEP use Week 13 interview (N = 66)			
Accepted PrEP at enrollment and started PrEP immediately	18 (72.0)	15 (78.9)	16 (72.7)
Delayed taking PrEP at Week 13 after initially accepting PrEP	2 (8.0)	3 (15.8)	1 (4.5)
Discontinued PrEP by Week 13 after taking PrEP for a period	1 (4.0)	0 (0.0)	0 (0.0)
Declined PrEP at Week 13	4 (16.0)	1 (5.3)	5 (22.7)
TFV‐DP levels at Week 13 (N = 54)			
Detectable TFV‐DP levels at Week 13	17 (85.0)	16 (88.9)	14 (87.5)
TFV‐DP levels ≥700 fmol/punch at Week 13	8 (40.0)	3 (16.7)	5 (31.3)
PrEP use at Week 26 interview (N = 63)			
Accepted PrEP between enrollment and Week 26 and continuing PrEP since prior visit	14 (56.0)	11 (68.8)	17 (77.3)
Delayed taking PrEP at Week 13 and Week 26 after initially accepting PrEP	2 (8.0)	2 (12.5)	0 (0.0)
Discontinued PrEP by Week 26 after taking PrEP for a period	3 (12.0)	1 (6.3)	1 (4.5)
Protocol‐defined PrEP product hold by Week 26	2 (8.0)	1 (6.3)	0 (0.0)
Continuously declined PrEP at Week 13 and Week 26	4 (16.0)	1 (6.3)	4 (18.2)
TFV‐DP levels at Week 26 (N = 54)			
Detectable TFV‐DP levels at Week 26	13 (68.4)	12 (66.7)	12 (70.6)
TFV‐DP levels ≥700 fmol/punch at Week 13	5 (26.3)	2 (11.1)	5 (29.4)

PrEP, pre‐exposure prophylaxis; TFV‐DP, tenofovir diphosphate levels detected in dried blood spot samples.

a Data are presented as median (interquartile range) for continuous variables and frequency (percentage) for categorical variables

b reflects data from the enrollment study visit.

All sites offered monthly, in‐person adherence clubs throughout study follow‐up. Among the 67 women in our sample, 60 (89.6%) attended at least one club with most attending an average of five clubs (IQR 3, 5). Of the 54 participants with dried blood spot data, 87.0% (N = 47) had detectable TFV‐DP levels at the Week 13 visit and 29.6% (N = 16) had TFV‐DP levels ≥700 fmol/punch. At Week 26, 68.5% (N = 37) had detectable TFV‐DP levels and 22.2% (N = 12) had levels ≥700 fmol/punch. These proportions were similar to the overall HPTN 082 sample 44.

### 3.2 Stigma experiences, PrEP disclosure and PrEP use

**Figure 1:**
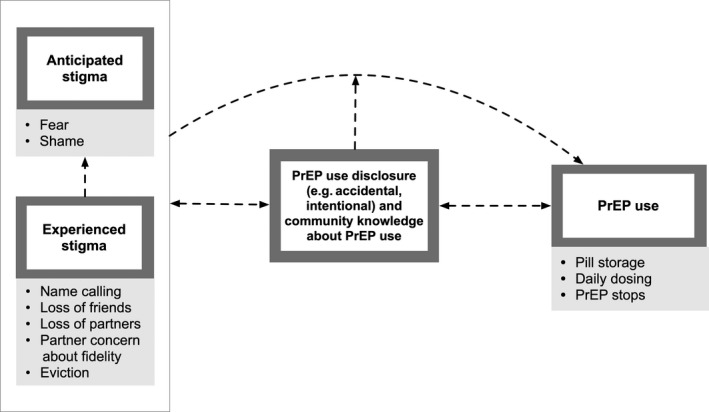
Key qualitative themes on the relationship among stigma, disclosure and PrEP adherence in the HPTN 082 sample.

Three themes emerged relating to: (1) stigma around PrEP use; (2) the negative influence of stigma on PrEP disclosure and adherence; and (3) disclosure as a strategy to combat stigma and improve PrEP use (Figure 1). We observed some differences in stigma and the role of disclosure in combatting stigma by study site and PrEP acceptance, but otherwise did not detect differences in themes by participant demographics (age, employment and living situation).

#### 3.2.1 Concerns and experiences with stigma around to PrEP use

AGYW described two main types of stigma related to PrEP use: HIV stigma arising when PrEP was mistaken for HIV treatment and sexual stigma when PrEP was thought to promote sexual promiscuity. Most participants described anticipated and experienced HIV stigma from someone seeing their pill bottles and assuming the participants were living with HIV. It was difficult for participants to explain that their pills were for HIV prevention rather than treatment:

“This pill bottle that they give us, it's the same bottle as those of ARVs, some people will think you are lying, she is taking the AIDS pills…it's not easy for a person to prove.” *Johannesburg participant, age 25, first interview, TFV‐DP at Week 13: 900 fmol/punch (4 to 6 doses/week)*

HIV stigma was feared and experienced from male partners, family members, and friends. Several participants reported that family and friends told them that they did not want to be associated with someone taking antiretroviral medications and a few participants described instances where they were asked to leave their homes or felt socially isolated because of their PrEP use. Participants also commonly said their male partners feared that their female partners' PrEP use would lead others to assume that they (the male partners) are HIV‐infected (regardless of male partner's HIV status). Some participants also said their male partners did not want them to use PrEP because their male partners would be teased for being with a woman who was taking HIV medications:

“When I started taking Truvada, my husband and his friends got in…When people left he said, ‘My friends were laughing at me that your wife has been taking ARVs.' It stirs quarrels in marriages…He said, ‘I am being labeled that I am sleeping with you and you are labeled that you have HIV.'” *Harare participant, age 20, first interview, discontinued PrEP prior to Week 13*

In addition to HIV stigma, participants also commonly discussed sexual stigma from PrEP use. They described how male partners, family members, and friends called them “whores” and “prostitutes” and how it was common for male partners to accuse them of having other sexual partners because of their PrEP use:

“Guys take it that you are this whore, you are sleeping around and that is why you are going to protect yourself. My ex‐boyfriend was saying I am a whore, why am I taking PrEP?” *Johannesburg participant, age 24, first interview, TFV‐DP at Week 13: 500 fmol/punch (2 to 3 doses/week)*

Most participants were concerned about how partners and others in the community would view their sexual behaviour because of their PrEP use, especially participants who described themselves as religious and those who lived with family who did not know they were sexually active. Participants who were in new relationships feared sexual stigma because their PrEP use could indicate to their primary partners that they had other sexual partners (“I wanted to tell him but I thought he is going to say I want to sleep around,” Cape Town participant, age 20, second interview, *TFV‐DP at Week 26: 300 fmol/punch, <2 doses/week*).

#### 3.2.2 Negative influence of stigma on PrEP disclosure, adherence and continuation

Stigma was mentioned in relation to PrEP disclosure at all study sites. Anticipated stigma prevented participants from disclosing PrEP use or resulted delayed disclosure.

“If I were to tell him that I am using PrEP he would not understand. He would shout, he would think that I have AIDS. So that is why I chose not to tell him and just leave [PrEP]. If I tell him I might as well just lose him.” *Cape Town participant, age 18, second interview, discontinued PrEP use between Week 13 and Week 26*

Many participants who did disclose their PrEP use experienced stigma from partners, family members, and friends; however, these negative disclosure experiences were more commonly mentioned during their first interview:

“The guy I was with dumped me for using PrEP…I showed him PrEP and he thought it was ARVs. He was saying I am cheating, why am I using these pills.” *Johannesburg participant, age 24, first interview*, *TFV‐DP at Week 13: 500 fmol/punch (2 to 3 doses/week)*

HIV and sexual stigma around PrEP use and related disclosure challenges were regularly described as barriers to PrEP acceptance and PrEP use, particularly early on in study participation. Anticipated stigma was a barrier to PrEP acceptance across all three study sites. Specifically, participants who declined PrEP or delayed PrEP use until their Week 13 interview discussed anticipated stigma more often than participants who accepted PrEP at enrollment:

“My mom would ask a lot of questions. She would think… now you wanna do things because you are taking PrEP. I feel like some people now think I can sleep with this one and this one…I don't want [to take it] because I don't wanna explain it.” *Johannesburg participant, age 24, declined PrEP at Week 13 and Week 26*

In addition, those who initially accepted PrEP but then discontinued at a later visit reported stigmatizing experiences that influenced them to stop taking PrEP. Several participants cited feelings of embarrassment about their pill bottles being seen or pills heard rattling and the teasing they experienced as a result (“that container would humiliate us in the street,” Harare participant, age 17, first interview). Some also said that they discontinued PrEP, refused PrEP during clinic visits, or did not carry their PrEP bottles when they traveled to avoid these negative experiences. For example, one Harare participant who discontinued PrEP during follow‐up said:

“I stopped taking [PrEP]. When I had taken them for some time, my pill bottle was seen by others and I was laughed at by others. That [comment], ‘She is taking ARVs’…it greatly affected me.” *Harare participant, age 23, first interview, discontinued PrEP prior to Week 13*

These links between negative stigma experiences and desires to delay, conceal, and/or stop PrEP use were seen across all three study sites.

#### 3.2.3 Disclosure as a strategy to combat stigma and improve PrEP use over time

During the first interview, participants commonly said they disclosed PrEP to only few people or did not yet disclose to anyone and many reported negative disclosure experiences which prevented them from taking PrEP, led to their hiding PrEP among people who they disclosed to, and discouraged them from telling others about PrEP. By their second interview, most participants had disclosed PrEP to a larger number of important individuals in their lives (sexual partners, family and/or friends) and participants cited positive disclosure experiences (e.g. the person they disclosed to understood and supported their desire to use PrEP) in large part due to increased confidence in disclosing, better understanding of PrEP and the differences between PrEP and HIV treatment, and support from staff and other participants. Many also described disclosure as a powerful way to combat stigma around PrEP in their households, increase community knowledge about PrEP, and improve their own PrEP use. Participants spoke about the importance of telling family and partners about PrEP before they found the pills and came to their own assumptions. This allowed participants to share their knowledge about PrEP while preventing rumors and stigmatizing experiences:

“I used to be scared to share with my relatives but I sat down with them to explain just like it is explained by the staff…because people who see the pills will not understand.” *Harare participant, age 23, second interview, TFV‐DP at Week 26: 700 fmol/punch (4 to 6 doses/week)*

Importantly, the relationships among stigma, disclosure and PrEP use differed by type of stigma and site. While participants commonly discussed the importance of improved disclosure skills and study brochures to help them combat HIV stigma around PrEP use, PrEP stigma related to being sexually active appeared more difficult to change. Participants in Cape Town and Johannesburg reported being able to avoid sexual stigma by explaining that PrEP was important for preventing HIV in case of sexual assault, which allowed them to disclose their PrEP use without being labeled as promiscuous:

“It was impossible for me to tell him at first…But I told him it wasn't about him. I can get raped…So me drinking the pill doesn't concentrate on him alone.” *Johannesburg participant, age 25, second interview*, *TFV‐DP at Week 26: 100 fmol/punch (<2 doses/week)*

However, we did not find similar explanations of PrEP disclosure to combat sexual stigma among Harare participants; several participants who experienced primarily sexual stigma reported stopping PrEP or poor PrEP adherence despite disclosure of PrEP use and changing community conversations around the HIV prevention benefits of PrEP:

“I'm surrounded by people who judge. It's the people who say, ‘You kids are naughty having sex before marriage, why [else] would you go for Truvada?' Having to take [PrEP] every day…I don't want to.” *Harare participant, age 19, first interview, declined PrEP at Week 13 and Week 26*

Positive disclosure experiences with family members, friends and sexual partners were linked with improved PrEP use for participants who were able to explain PrEP and its HIV prevention benefits, as these young women were more likely to then receive support from these others who acknowledged the importance of the participants’ PrEP use. These supportive individuals often helped remind and encourage them to take their daily pills. For example:

“I explained it to [people at home]. They ended up encouraging me. For example, when it's time if I am outside they call me and say, ‘Come it's time, your phone alarm is ringing.'” *Harare participant, age 17, second interview, TFV‐DP at Week 26: 800 fmol/punch (4 to 6 doses/week)*

Disclosure also helped improve PrEP adherence because participants were no longer embarrassed about carrying pills and taking PrEP in front of others:

“I was telling [my friends] about PrEP, explaining the study. I wanted to feel comfortable whenever taking the pill even if I am with them. They are not saying anything bad and they support me.” *Johannesburg participant, age 25, second interview, TFV‐DP at Week 26: 2100 fmol/punch (7 doses/week)*

In addition to increased PrEP disclosure to household members by their second interview, many participants talked about proactively discussing PrEP more broadly, in their communities. For example, one participant referred to herself as a “PrEP ambassador,” and described how she shared knowledge about PrEP in her community (“I want to be the change…I want to announce PrEP so that many can know about it,” Cape Town participant, age 22, first interview, TFV‐DP at Week 13: 600 fmol/punch, 2 to 3 doses/week). Some participants felt that broader disclosure of PrEP use and more widespread knowledge about PrEP could help reduce PrEP stigma in their communities.

### 3.3 Intervention opportunities to combat stigma and improve disclosure skills

**Figure 2:**
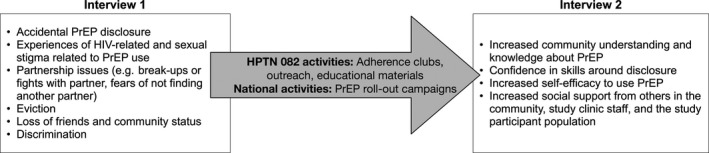
Changes in stigma, disclosure and PrEP experiences over time attributed to HPTN 082 intervention and national activities.

Participants discussed several HPTN 082 activities that helped change their stigma experiences, improve their confidence in disclosure skills, and increase PrEP disclosure and use over time, suggesting that these are potentially successful interventions to combat stigma and facilitate PrEP adherence (Figure 2). Participants attributed changes in stigma and disclosure skills to (1) HPTN 082 counselling sessions where they could discuss disclosure strategies and role play disclosure with a counsellor, (2) outreach materials (e.g. informational brochures, t‐shirts about PrEP) which they could give to others to explain PrEP, (3) support from staff, and (4) community outreach campaigns led by staff:

“I am now dignified…Now no one shouts a thing if I pass by. I thank the T‐shirts and the [study staff] here. They help explain PrEP to the community and that is how they understood.” *Harare participant, age 23, second interview, TFV‐DP at Week 26: 700 fmol/punch (4 to 6 doses/week)*

Participants also mentioned the value of discreet pill carrying cases provided to study participants which looked like cosmetic cases. These cases allowed them to conceal PrEP tablets and reduced rattling and unintentional disclosure. Participants also described the HPTN 082 monthly adherence clubs as an opportunity to share their challenges with stigma, disclosure, and PrEP use and to receive peer support:

“At adherence clubs we meet, share our experiences. If I share that I was laughed at by people saying I have AIDS someone will say, ‘No, being laughed at is common, just ignore it.’ We are giving each other advice. When you are home you will still be in pain that people laughed at you and can think of stopping the pill. But you hear someone encouraging you.” *Harare participant, age 23, second interview, TFV‐DP at Week 26: 700 fmol/punch (4 to 6 doses/week)*

National and local PrEP campaigns, particularly in South Africa, included radio and television advertisements and community outreach campaigns about PrEP and were seen by participants as helping to change knowledge and awareness of PrEP and reduce HIV stigma around product use. Participants in turn associated these activities with improvements in their PrEP use:

“People ended up believing that this thing about PrEP is true. They saw it on magazines. And there are advertisements with PrEP on it and that is how they understood it.” *Cape Town participant, age 19, second interview, TFV‐DP at Week 26: 200 fmol/punch (<2 doses/week)*

## 4 Discussion

In this qualitative analysis among AGYW participating in the HPTN 082 study, participants described PrEP stigma related to HIV and sexual activity. Stigma was a barrier to disclosure and PrEP use particularly during early study participation. Over the time between interviews, women became more comfortable disclosing PrEP to those around them. Study‐supported activities to improve disclosure skills, social support and PrEP visibility were cited as helping to reduce stigma and improve PrEP adherence. However, participants who primarily experienced sexual stigma seemed to have more difficulty disclosing product use and maintaining PrEP use. Results from this study highlight the importance of PrEP providers and counsellors acknowledging difficulties with stigma and product disclosure and developing interventions to empower young women around PrEP use, particularly early on when they are first considering PrEP for HIV prevention. Our findings also underscore the need to move towards multi‐level PrEP programmes (e.g. media campaigns, community outreach) which have the potential to change norms about sexuality and increase community knowledge around PrEP.

While this work is unique in its focus on PrEP stigma among African AGYW, our findings are largely consistent with PrEP studies with African adults 7, 18, 28, 45. Women in South Africa have cited support from male partners and family members as a facilitator of PrEP use, and have described PrEP stigma, relationship power dynamics and issues around product disclosure as barriers to PrEP use 45. Similar concerns about stigma and disclosure have been described in programmatic PrEP delivery settings among heterosexual HIV serodiscordant couples in Nigeria and MSM in the United States, particularly when PrEP is explicitly marketed for “high‐risk populations” 14, 46, 47. Mediation analyses among MSM and transgender women in the United States have shown that stigma has a direct negative association on PrEP adherence and an indirect influence on PrEP use through fears about disclosure, highlighting the importance of both stigma and disclosure on regular PrEP use 22, although similar analyses are needed among African AGYW.

Longitudinal qualitative data collection allows evaluation of dynamic changes in narratives around stigma, disclosure and PrEP use. Initial guidelines to target PrEP for FSWs could have affected participants’ attitudes about PrEP use, and contributed to stigma about sexual promiscuity and PrEP. During implementation of HPTN 082, PrEP programmes in South Africa and Zimbabwe expanded to include AGYW who were not FSWs in urban and peri‐urban centers accompanied by local demand‐creation activities (e.g. radio and television advertisements). Increasing numbers of PrEP users and word‐of‐mouth information about PrEP created local pockets of PrEP awareness among AGYW in our study settings which may have influenced stigma and disclosure experiences 20, 48, 49. Expanding efforts to create demand around PrEP and integrate PrEP within primary care and sexual and reproductive health services in both countries also gained traction during the study period and will likely continue to increase awareness and acceptability of PrEP 50, 51, 52, 53.

These results highlight the opportunity for PrEP programmes to improve PrEP uptake, continuation, and effective use by addressing stigma around PrEP and concerns around disclosure. In‐person and social media campaigns that brand PrEP around wellness and empowerment, rather than HIV risk and sexual behaviour, have the potential to counter stigmatizing narratives around PrEP 20, 54, 55. Sexual stigma among AGYW is likely more difficult to change than HIV‐related stigma. PrEP programmes need positive messaging about PrEP as a cornerstone of sexual and reproductive health in settings of generalized HIV risk, including the less stigmatizing message that PrEP is appropriate for HIV prevention due to age and geography rather than individual behaviour. Youth‐competent services, adolescent‐friendly and de‐medicalized PrEP delivery approaches (e.g. online PrEP ordering, pharmacy‐based delivery), and PrEP delivery in family planning and reproductive healthcare clinics can change perceptions that PrEP is only for individuals with risky sexual behaviour 52, 56, 57, 58, 59, 60. Social support interventions are likely key strategies to improve PrEP effectiveness among youth and in‐person adherence clubs have been found to improve ART adherence among adolescents living with HIV 61. In HPTN 082, peer adherence clubs provided opportunities for AGYW to discuss stigmatizing experiences and learn empowering skills around PrEP disclosure. However, attending these meetings is not feasible for all and PrEP delivery settings may consider offering adherence club content digitally (e.g. via a WhatsApp platform) to reduce barriers related to in‐person club attendance. This approach for scalable adherence club delivery is currently being tested in an ongoing PrEP trial.

The strengths of this study include the large sample size from three sites, longitudinal data collection, and high retention between interviews. We had a multi‐national team of coders who provided context‐specific perspectives on the data. This study also had a number of limitations. We relied on self‐reported information about PrEP use during the interviews which may be biased. In our results, we also report participants’ TFV‐DP levels and the approximate number of PrEP doses per week based on established drug concentration thresholds 38, but these thresholds were determined in studies conducted in the United States with men and women and may not be the same for young African women. Women discussed changes in disclosure and stigma experiences as a facilitator of improved PrEP use over time, but it is also possible that this was a bidirectional relationship whereby women reduced their PrEP use over time and therefore experienced less stigma and fewer negative disclosure experiences. Although women attributed positive changes in stigma and disclosure experiences to study‐related community outreach and HPTN 082 adherence clubs, it is not possible to disentangle the influence of these study activities from ongoing PrEP roll‐out efforts occurring in South Africa and Zimbabwe (e.g. radio and television advertisements). While we focused on within‐participant changes between the Week 13 and Week 26 interviews, it is also likely that there were important temporal trends in community norms and perceptions of PrEP as a result of ongoing PrEP roll‐out rather than study activities. The qualitative participants may have been highly motivated to continue in the study, engage in adherence clubs, and use PrEP and results may not be generalizable to AGYW seeking PrEP in programmatic delivery settings.

## 5 Conclusions

South African and Zimbabwean AGYW described experiences of PrEP stigma related to both HIV and sexual behaviour, particularly early on after initiating PrEP, and discussed how stigma negatively influenced their PrEP disclosure, uptake and adherence. For those experiencing stigma, PrEP disclosure became a tool for changing family and community beliefs around PrEP and improving PrEP use. Changes in stigma, disclosure experiences and PrEP use over time highlight opportunities for future PrEP programmes to improve PrEP effectiveness through demand creation and community education campaigns, empowerment and social support interventions, and adolescent‐friendly healthcare services, with the ultimate goal of shifting social norms around HIV prevention and sexual behaviour for AGYW.

## Competing interests

The authors report no conflicts of interest.

## Authors’ contributions

CC, SDM, SH, LGB, NM and MC designed the parent study. JV, NK, FS, CC, SDM, SH, LGB, NM and MC designed the qualitative study and analysis. JV conducted all analyses and wrote the manuscript. All authors reviewed and approved the final version of the manuscript. JV contributed to qualitative study design, qualitative coding, results interpretation and drafted the manuscript. NK contributed to qualitative study design, qualitative data collection and coding, results interpretation, and edited the manuscript. FS contributed to qualitative study design, qualitative coding, results interpretation and edited the manuscript. MC, PM, KM, NM and LN contributed to data collection, qualitative coding and edited the manuscript. MAB and MA contributed to qualitative coding and edited the manuscript. SH, LGB, NM and MC contributed to study design, results interpretation and edited the manuscript. RH contributed to results interpretation and edited the manuscript. CC and SDM contributed to funding, study design, result interpretation and edited the manuscript.
